# Maternal Psychological Well-Being as a Protector in Infantile Colic

**DOI:** 10.3390/nu16142342

**Published:** 2024-07-19

**Authors:** Victoria Eugenia Garnacho-Garnacho, Elena Sonsoles Rodríguez-López, Ángel Oliva-Pascual-Vaca, Leire Goenaga-Echave, Álvaro Otero-Campos

**Affiliations:** 1 Physiotherapy and Health Research Group (FYSA), Department of Physiotherapy, Faculty of Health Sciences—HM Hospitals, University Camilo José Cela, 28014 Madrid, Spain; vegarnacho@ucjc.edu (V.E.G.-G.); aotero@ucjc.edu (Á.O.-C.); 2 Department of Physiotherapy, Faculty of Health Sciences—HM Hospitals, University Camilo José Cela, 28014 Madrid, Spain; leiregoe@gmail.com; 3 Instituto de Biomedicina de Sevilla (IBiS), Departmento de Fisioterapia, Universidad de Sevilla, 41013 Seville, Spain

**Keywords:** postpartum depression, anxiety disorder, functional gastrointestinal disorders, infantile colic, infants, breastfeeding

## Abstract

(1) Background: Infantile colic (IC) is a functional gastrointestinal disorder that affects around 20% of infants, and postpartum (PPD) depression is a common disorder that affects between 15 and 22% of mothers. In this study, our objective was to evaluate the relationship between the maternal psychological state in the first postpartum year and IC, with the aim of assessing the importance of feeding type in infants and maternal well-being. (2) Methods: A cross-sectional study was conducted in women in their first year postpartum. Demographic, medical, and obstetric data of the mothers and infants were collected, and the type of feeding was identified. The emotional status of the mother was evaluated using the Edinburgh Postnatal Depression Scale (EPDS), and the Infant Colic Severity Questionnaire (ICSQ) was used for IC diagnosis. (3) Results: A total of 528 women were analyzed, of which 170 (32%) were diagnosed with possible PPD. Two-thirds of the women without depression breastfed their babies on demand; therefore, we report that exclusive breastfeeding (EBF) appears to reduce the risk of possible PPD (*p* < 0.001; OR = 2.353). IC was present in 39% of babies, and around 70% of babies without colic were breastfed on demand. Infants who were not exclusively breastfed showed almost double the risk of developing colic (*p* = 0.016; OR = 1.577). There was a significant association between the EPDS and ICSQ scores (*p* < 0.001). More than half of the women with PPD had babies with colic. However, our results show that 75% of babies without colic had mothers who reported optimal postpartum emotional well-being (*p* < 0.001; OR = 2.105). (4) Conclusions: The results of this study suggest that postpartum maternal psychological well-being reduces the risk of IC. Therefore, we report that EBF on demand, together with a healthy emotional state in new mothers, may be a protective factor against colic in infants.

## 1. Introduction

Microbial settlement of the newborn’s gut is one of the most crucial biological occurrences in existence. In recent times, microorganisms have been identified in the prenatal milieu, including the amniotic fluid, umbilical cord blood, fetal membranes, placenta, and meconium, challenging the widely accepted notion of an aseptic fetal digestive system [1,2,3]. In the first seven days post-birth, bacterial clusters such as Staphylococcus, Enterococcus, Streptococcus, and members of the Enterobacteriaceae family attain high population concentrations, fostering a reduced environment conducive to the establishment of strict anaerobes [4]. Over the past few decades, numerous medical research studies have explored factors that might affect this colonization phase, distinguishing the method of birth, the nutrition type, and the administration of antimicrobials as the most impactful elements [3].

The role of the childhood intestinal microbiota in gastrointestinal disorders is unknown. Infantile colic (IC) is a functional gastrointestinal disorder (FGID) that affects around 20% of infants [5]. Currently, the etiology of IC is based on some physiological and psychological hypotheses [6]. In this framework, rather than being merely classified as motility disorders, FGIDs are characterized as neuro-gastroenterological conditions involving and modifying brain–gut interactions, particularly the brain–gut axis [7]. Just as infantile colic can cause depression, anxiety, and stress in the mother, mental health disorders can cause the development of IC by affecting the baby [8]. The relationship between the baby and the mother can be negatively affected by factors such as depression, anxiety, and stress, which are very common during the postpartum period [9].

IC affects the health of the infant and also the daily life of the family. Currently, the main management of IC should provide complete and updated parental education, reassurance, and nutritional advice because it lowers the quality of life of mothers [10]. Therefore, our objective was to evaluate the relationship between the maternal psychological state in the first postpartum year and IC, with the aim to assess the type of feeding and other factors in infants and maternal well-being.

## 2. Materials and Methods

A cross-sectional study was conducted in women in their first year postpartum. This study was approved by the Ethics Committee of the University Camilo José Cela (18_23_VIR, 26 February 2024; Madrid, Spain). All women received detailed information about the research and written informed consent before commencing.

Postpartum women were selected from outpatient clinics (primary care settings, midwifery centers, pediatric consultations, and physiotherapy centers) and breastfeeding groups in Madrid. The sample size was calculated using the G*power software version 3.1.9.6 (Kiel University, Kiel, Germany). Then, a two-tailed hypothesis with an effect size d of 0.5, an α error probability of 0.05, and a statistical power of 0.99 was employed for the sample size calculation. The prevalence of probable depression at 2 months, 6 months, and 1 year postpartum, using a cut-off point ≥ 10 on the Edinburgh Postnatal Depression Scale (EPDS) [11], was 30.3%, 26.0%, and 25.3%, respectively. Thus, the estimated minimum sample size was 394 postpartum women consisting of 99 with a diagnosis of postnatal depression and 295 without.

Data were collected between February and May 2024 through an anonymous online questionnaire using the Survey Monkey^®^ platform (San Mateo, CA, USA). We invited participants to complete the questionnaire, and their information was automatically processed and protected on a server with a password. The data were stored by the research team in accordance with the legislation. Participants did not receive any remuneration for their participation in this study.

The study sample comprised women in their first year postpartum, who were divided into the following two groups: women with probable postpartum depression (PPD) according to the EPDS and women without PPD. The exclusion criteria were as follows: (1) diagnosis of mental illness prior to pregnancy; (2) being under 18 years of age; (3) an inability to adequately understand instructions in Spanish; (4) extremely preterm infants (less than 28 weeks gestational age) [11] or who had suffered any intracranial hemorrhages; (5) infants diagnosed with a pathological illness; and (6) allergies or food intolerances.

Data were collected through an online survey supervised by researchers. Before circulating the survey to the participants, its usability and performance were tested and verified. The women were instructed to review their responses before final submission. The questionnaire contained four main sections: (1) anthropometric and educational data; (2) medical history, obstetric data, and type of feeding; (3) the Infant Colic Severity Questionnaire (ICSQ); and (4) the EPDS.

Maternal variables were age (in years), weight, height, body mass index (BMI), medical history, and maternal education level. Lifestyle factors were collected such as alcohol consumption, smoking, being physically active (minutes walked per day and days per week were self-reported, as well as frequency of sports practice), and levels of body fat (BMI was calculated using weight in kilograms (kg) divided by the square of body height in meters (m), and the units were kg/m^2^). The obstetric data collected were the date of birth, type of delivery (cesarean or vaginal), and parity (primiparous or multiparous). The following infant data were also collected: weeks of gestation, birth weight, intrapartum complications, fetal distress, diagnosed diseases, type of feeding, and allergies or food intolerances.

The psychological status of the mother was evaluated using the EPDS, which is a tool specialized for screening depression in pregnant and postpartum women [12] and validated for Spanish [13]. The scale [14] is a 10-item self-report questionnaire (range 0–30).

The ICSQ was used for IC diagnosis and evaluation [15], as it is the most well-known, reliable, and validated questionnaire. A total of 25 questions were completed by the mothers, which evaluated crying, sleep, suckling, stools, burping, vomiting, and gas. The highest score was 100; scores over 50 points were considered to indicate that an infant suffered from IC.

The IBM Statistics Package for Social Science, v.26 (IBM Corp, New York, NY, USA), was used to analyze the data, which are provided as the mean, standard deviation, and percentages. The Shapiro–Wilk test was used to check the normality of data in the sample. Between the presence of depression or colic, the means were compared using the Student t-test, and proportions using the Chi-squared test. Maternal education level and birth weight were analyzed using the Monte Carlo simulation. The estimated odds ratio (OR) and 95% confidence interval (CI) for the OR were analyzed through the risk estimate of crosstabs. Bivariate correlations among quantitative variables were assessed using Pearson’s coefficient. A *p*-value of <0.05 was considered statistically significant with a confidence level of 95%.

## 3. Results

A total of 854 women were invited to participate in this study and were provided access to the questionnaire; however, 178 women did not complete the questionnaire. A total of 148 women were excluded for the following reasons: mental illness prior to pregnancy (*n* = 25); postpartum more than one year (*n* = 68); premature babies (*n* = 15); infants diagnosed with pathological illness (*n* = 6); and food intolerances (*n* = 34). Therefore, the final sample for the analysis consisted of 528 women in their first year postpartum.

The mean age of the mothers was 35.36 ± 3.98 years; a total of 68.1% of mothers had normal weight, 18.7% overweight, and 8.6% obesity; approximately 80% of the women were university graduates; and this was the first baby for 64.9% of the women. More than half of the babies were less than 6 months old (*n* = 353). Generally, the babies had birth weights appropriate for gestational age (98.9%), and most infants were born full-term (95.2%). Only one-third of infants were born via cesarean section. Most of the babies (*n* = 228, 64.5%) within 6 months of age were exclusively and on-demand breastfed. In the babies older than 6 months, 80% were still breastfed in addition to complementary feeding. Anthropometric variables, maternal education level, lifestyle, obstetric data, and data of infants are provided in Table 1 and Table 2, respectively.

One hundred and seventy women were diagnosed with possible PPD through the self-reported EPDS, which represents 32.2%. Depression was significantly lower in mothers with high education levels (*χ*^2^ [3] = 8.652; *p* = 0.024). Totals of 71.6% and 24.1% of the women with depression and 83.2% and 14.1% of the women without depression had university and secondary education, respectively. Data related to age, BMI, lifestyle, or birth were not different in women with PPD (Table 1). Around 75% of the women without depression breastfed their babies on demand; this result indicates that exclusive breastfeeding appears to reduce the risk of possible PPD (*χ*^2^ [1] = 20.227; *p* < 0.001; OR = 2.353, CI95% = 1.614–3.43).

IC was present in 39% of babies (*n* = 206). The most common symptoms were regurgitation (*n* = 375, 71.4%), daytime (after 7 p.m.) infant crying (*n* = 241, 45.6%), and non-stop crying *(n* = 142, 26.9%). Colic was more present at 1–2 months (*n* = 63, 30.6%) and 3–5 months of age (*n* = 68, 33%) (*χ*^2^ [4] = 11.320; *p* = 0.045). Variables related to the mother, such as age, BMI, maternal education level, or lifestyle, did not influence the colic group. Half of the babies of primiparous women had colic (*n* = 146); however, only one-third of the women with more than one child had babies with colic (*χ*^2^ [1] = 5.844; *p* = 0.020). Data related to birth (gestation weeks, delivery, or birth weight) did not influence the colic group (Table 2). Around 70% of babies without colic were exclusively breastfed on demand; the results suggest that babies who are not exclusively breastfed have almost double the risk of developing colic (*χ*^2^ [1] = 6.091; *p* = 0.016; OR = 1.577, CI95% = 1.097–2.266).

**Figure 1:**
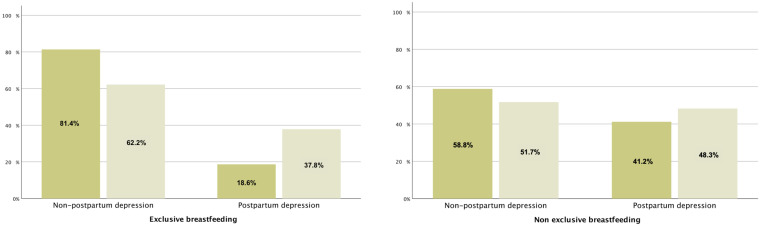
Relationship between infantile colic and postpartum depression according to type of feeding. The graph represents absence of infantile colic (dark brown) and presence of infantile colic (light brown).

Infants with colic had mothers with higher EPDS scores (*p* < 0.001). The PPD scores were also higher in the presence of colic (*p* < 0.001). There was a significant association between the EPDS and ICSQ scores (r = 0.265; *p* < 0.001). More than half of the women with PPD had babies with colic. However, 75% of babies without colic had mothers who reported optimal postpartum emotional well-being (*χ*^2^ [1] = 15.585; *p* < 0.001; OR = 2.105, CI 95% = 1.450–3.056) (Figure 1).

## 4. Discussion

Evidence from this research suggests that postpartum maternal psychological well-being reduces the risk of infantile colic. In our study, the results indicate that exclusive breastfeeding may reduce infantile colic and depression.

PPD is a common disorder affecting between 15 and 22% of Spanish mothers [16]. Moreover, this disorder is considered a public health problem [17]. Our data showed a slightly higher frequency of PPD than the 32.2% reported earlier. The high prevalence of undiagnosed psychological disorders represents a significant challenge for public health. Individuals who do not receive an adequate diagnosis are at risk of experiencing a progression of their symptoms, which can lead to more severe and difficult-to-manage health problems. This phenomenon also has implications for health policy, as it highlights the need to improve early detection and treatment systems for these disorders [18,19,20,21,22]. Mental aspects may affect bodily functions and emotional well-being, having a beneficial impact on physical health [23].

The incidence of PDD depends on several factors, such as age, BMI, education level, and type of delivery, among others. Moreover, high maternal age has been reported to increase the risk of depression [24]. In our research, no differences were found in terms of gestational age, perhaps because more than 90% of the women were over 30 years old. People who suffer from chronic illnesses are at a higher risk of developing mental illnesses [25]. For example, research indicates that individuals with chronic illnesses and depression tend to experience more severe symptoms of both conditions. This co-occurrence can exacerbate the course and severity of each disease, creating a detrimental cycle for the patient [26]. Mental illnesses are not only a consequence of chronic diseases but can also be a predisposing factor for the development of various chronic conditions. Studies indicate that people suffering from depression have a higher risk of developing heart disease, diabetes, stroke, chronic pain, osteoporosis, and Alzheimer’s disease [27]. On the other hand, clinically depressive symptoms appear to be associated with visceral adiposity and its relation to total fat in women [28]. However, our data did not report this association. We believe that the absence of this association in our data is because only 8.6% of the women in our study had obesity measured through BMI, and visceral adiposity was not controlled with other measurements as in other studies.

Targeting lifestyle behaviors during pregnancy is relevant to preventing PPD and its consequences [17]. Exercise could reduce PPD [29,30,31], but this affirmation is controversial. Our findings were similar to those of previous research [17,31] that did not show any effect of exercise on postnatal depression. The consumption of alcohol or tobacco also did not show a significant association in women with postpartum depression. Moreover, we did not collect data about maternal nutrition; therefore, it would be interesting for future studies to investigate these factors because maternal nutrition might influence the development and course of postpartum depression [32].

Our findings suggest that depression is lower in mothers with high educational levels. However, other authors have reported depression to be lower in mothers with lower educational levels [33]. These authors speculated that greater awareness at higher educational levels may lead to greater anxiety, which was questioned in the Edinburgh study. This result was not demonstrated in our research, perhaps because 80% of our sample were university graduates. Almost all university mothers chose exclusive breastfeeding (EBF), and one study showed that educating mothers about breastfeeding is more important than the mother’s level of education for the maintenance of EBF [34].

Breastfeeding is strongly recommended by the World Health Organization (WHO) [35] because it has significant benefits for the baby, the mother, and even public health. Therefore, the cessation of breastfeeding has a negative impact on child health [36,37,38]; it has also been associated with an increased risk of PPD [39]. Our results were along the same lines, suggesting that EBF on demand may be a protective factor for the emotional state of new mothers. The widespread knowledge that women’s physical and mental health [40] are negatively affected by the cessation of breastfeeding should prompt the promotion of breastfeeding and postpartum maternal care.

Maternal separation and lack of contact in the first years of life due to postpartum depression affect the baby’s neuroendocrine and behavioral reactions [40]. The skin is the largest organ in the body with different functions [41]. The skin is innervated by afferent sensory nerves, which detect and distinguish between harmless and noxious stimuli, and efferent autonomic nerves, which maintain homeostasis of the barrier tissue. The discovery of unmyelinated C fibers, also known as tactile C fibers, is fundamental to understanding how caressing contact between mothers and their children through the skin achieves great benefits [42]. Touch performed at optimal stroking speeds on tactile C fibers [43,44] reduces heart rate in full-term and preterm infants [45] and pain responses in full-term infants undergoing painful medical procedures [46]. On the contrary, negative emotions processed by the brain can drive changes in the intestinal environment and alter microbial composition [47]. Maternal mental health significantly impacts the composition and functioning of infants’ intestinal microbiota [48]. Children’s intestinal microbiota is influenced not only by genetic and environmental factors but also by maternal mental health and microbiota [49]. During pregnancy and lactation, mothers transfer microbiota to their children, which can affect intestinal development and the child’s immune system [50]. Mental health issues in the mother can disrupt this microbiota transfer, potentially affecting child development and predisposing children to physical and mental health problems [48]. The interrelationships between maternal mental health, intestinal microbiota, and infant intestinal health have profound implications for child development. Children exposed to altered microbiota due to maternal mental health issues may have a higher risk of developing gastrointestinal, immunological, and mental health disorders [51].

This underscores the importance of addressing maternal mental health as an integral component of child health. To mitigate these risks, we suggest interventions that promote maternal mental health, such as psychological support programs and stress-reduction strategies. Additionally, promoting a healthy diet that supports balanced intestinal microbiota can benefit both the mother and child [52].

The prevalence of FGID in infants/toddlers is 27.1% (0–3 years old) and 67–87% (2–4 months of age) [53]. Our research analyzed only IC, with the results showing that 39% of babies had colic, which is slightly higher than other reported data. However, when infants were 1–2 months of age, the prevalence of FGID increased by around 50%, which was less than data reported in other research. In our study, the most common disorders in infants were regurgitation (25.9%), colic (5.9%), and functional constipation (4.7%) [53]. We also found regurgitation to be the most frequent symptom, followed by crying. Infant data, such as gestation weeks or birth weight, or maternal data, such as age, BMI, education level, or lifestyle, appear to not influence the development of infantile colic. However, colic appears to occur less in infants of mothers with experience caring for other children. We also did not find an association between colic and vaginal births/cesarean sections, although the influence of the mode of delivery on the infant microbiome has been widely studied [54]. We found that around 70% of babies without colic were breastfed on demand. The results of our study suggest that babies who were not exclusively breastfed have almost double the risk of developing colic. Breastfeeding may also have an impact on microbiota. Substantive evidence shows early nutrition influences the development of the microbiome and immune system, affecting lifelong health [55]. 

The synthesized data produced by this study show that maternal psychological well-being reduces the risk of IC. The effect of the mother’s psychological state and behavior are also reported in the short term [33] and long term [56]. Because of the maternal depressive state, the balance of the microbial composition can be affected, producing an increase in a pro-inflammatory microbial profile and increasing specific microorganisms, such as Veillonella ratti, Anaerobutyricum hallii, and Roseburia, which are known to produce gases in the intestines and are related to the microbial profile of babies with colic [57]. This can cause increased intestinal gas and discomfort, which can contribute to colic symptoms. In addition, PPD and anxiety are associated with early breastfeeding cessation (i.e., stopping breastfeeding before 2 months) [58]. As demonstrated in other studies, we found that both IC and prolonged crying are associated with higher scores on maternal depression scales [59]. Furthermore, it was found that as mothers’ depression scores increased, their maternal attachment scores decreased [5]. Therefore, health professionals must prioritize the daily provision of pre- and postnatal maternal support based on scientific evidence. For this reason, knowledge of the importance of early screening for postpartum depression symptoms is necessary to promote the health of the mother–infant dyad. Moreover, early implementation of psychological interventions within the postpartum period could improve the health of both mother and infant.

### Study Strengths and Limitations

First, a selection bias may exist in this study, even though the research was conducted on a large sample. The sample was selected through outpatient clinics (primary care settings, midwifery centers, pediatric consultations, and physiotherapy centers) and breastfeeding groups in Madrid. On the other hand, our questionnaires were answered once via self-report, with a cross-sectional design; thus, we were unable to obtain data on maternal psychological evolution, colic, or breastfeeding throughout the entire postpartum period.

Finally, one of the main limitations of our study lies in the difficulty of controlling subjects’ mental health throughout their entire lives. Variations in access to mental health services, the accuracy of self-reports, and the influence of uncontrolled external factors make it challenging to achieve comprehensive and continuous monitoring of mental health [60]. Despite utilizing multiple data sources to mitigate these limitations, we acknowledge that achieving complete control is practically unattainable. One of the greatest obstacles is the variability in access to mental health services [61]. Future research should focus on developing and evaluating holistic interventions that address both mental and biological health, benefiting mothers and children alike.

Furthermore, individuals may experience differences in the quality and availability of mental and biological health services over time, introducing inconsistencies in the collected data. Additionally, the accuracy of self-reports may be affected by factors such as stigma associated with mental disorders and variability in self-assessment.

To address these limitations, we propose that future research should focus on developing longitudinal studies. These studies would allow for more detailed and prolonged monitoring of subjects’ mental health, providing more reliable and comprehensive data. Furthermore, the integration of advanced monitoring technologies, such as mobile applications and wearable devices, could offer more precise and continuous methods to assess mental health, helping to overcome some of the current limitations.

## 5. Conclusions

The results of this study suggest that postpartum maternal psychological well-being reduces the risk of infantile colic. Furthermore, exclusive breastfeeding on demand may be a protective factor for the emotional state of new mothers and colic in infants. To fully understand the dynamics between mothers and infants, broader research is required to investigate integrated approaches that observe maternal and infant factors together.

## Figures and Tables

**Table 1:** Anthropometric data, educational level, lifestyle, and obstetrics of the mothers via the ICSQ and EPDS scores.

	Total Group (*n* = 528)		Postpartum Depression					Infantile Colic				
			Present (*n* = 170)		Absent (*n* = 358)		*p*-Value	Present (*n* = 206)		Absent (*n* = 322)		*p*-Value
	Mean	SD	Mean	SD	Mean	SD		Mean	SD	Mean	SD	
Age (years)	35.36	3.98	35.21	3.98	35.43	3.98	0.566 ^a^	35.29	3.68	35.40	4.16	0.752 ^a^
BMI (kg/m^2^)	23.74	4.68	23.76	4.37	23.74	4.83	0.956 ^a^	23.91	5.02	23.64	4.46	0.515 ^a^
Maternal education level												
Illiterate/literate (n. %)	1	0.2%	0	0	1	100%	0.024 ^b^	1	100%	0	0	0.461 ^b^
Elementary school (n. %)	13	3.2%	6	46.2%	7	53.8%		4	30.8%	9	69.2%	
Secondary school (n. %)	75	28.5%	34	45.3%	41	54.6%		7	35.0%	13	65%	
University (n. %)	343	79.4%	101	29.4%	242	70.6%		159	40.0%	239	60%	
Lifestyle												
Smoke (Yes (n. %))	26	4.9%	11	42.3%	15	57.7%	0.529 ^b^	11	42.3%	15	57.7%	0.392 ^b^
Alcohol (Yes (n. %))	2	0.4%	1	50%	1	50%	1.000 ^b^	0	0%	2	100%	0.082 ^b^
Walking												
Hours/day	2.31	0.70	2.31	0.78	2.31	0.66	0.942 ^a^	2.40	0.77	2.25	0.64	0.017 ^a^
Days/week	5.28	1.78	5.15	1.84	5.34	1.74	0.263 ^a^	5.29	1.74	5.28	1.80	0.921 ^a^
Sports practice												
Hours/day	1.69	0.76	1.66	0.86	1.71	0.71	0.551 ^a^	1.69	0.75	1.69	0.77	0.914 ^a^
Days/week	2.50	1.61	2.28	1.61	2.60	1.60	0.057 ^a^	2.57	1.73	2.47	1.53	0.523 ^a^
Obstetrics data												
Postpartum days	149.20	96.22	165.81	102.46	141.30	92.22	0.006 ^a^	142.78	95.36	153.30	96.70	0.221 ^a^
Parity												
Primiparous (n. %)	341	64.6%	229	67.2%	112	32.8%	0.698 ^b^	146	42.8%	195	57.2%	0.020 ^b^
Multiparous (n. %)	187	35.4%	58	31%	129	69%		60	32.1%	127	67.9%	
Number of children	1.4	0.59	1.36	0.53	1.42	0.628	0.356 ^a^	1.33	0.53	1.45	0.63	0.022 ^a^
ICSQ score	47.32	8.16	49.90	9.19	46.09	7.32	<0.001 ^a^	55.37	4.95	42.17	5.04	<0.001 ^a^
EPDS score	9.54	5.36	15.76	3.54	6.59	3.06	<0.001 ^a^	11.24	5.69	8.45	4.85	<0.001 ^a^

SD—standard deviation; kg—kilograms; ICSQ—Infant Colic Severity Questionnaire; and EPDS—Edinburgh Postnatal Depression Scale. The significance level was set at *p* < 0.05. (^a^) Difference between groups according to the *p*-value based on the Student *t*-test. (^b^) Proportions were analyzed using the Chi-squared test.

**Table 2:** Age, birth data, and feeding of the infant.

	Total Group (*n* = 528)		Postpartum Depression					Infantile Colic				
			Present (*n* = 170)		Absent (*n* = 358)		*p*-Value ^a^	Present (*n* = 206)		Absent (*n* = 322)		*p*-Value ^a^
	Mean	SD	Mean	SD	Mean	SD		Mean	SD	Mean	SD	
Age groups												
Less than 1 month	48	9.1%	12	25%	36	75%	0.089	15	31.3%	33	68.8%	0.045
1–2 months	128	24.2%	37	28.9%	91	71.1%		63	49.2%	65	50.8%	
3–5 months	177	33.5	51	28.8%	126	71.2%		68	38.4%	109	61.6%	
6–8 months	95	18%	36	37.9%	59	62.1%		29	30.5%	66	69.5%	
9–12 months	79	15%	33	41.8%	46	58.2%		30	38%	49	62%	
Birth data												
Mode of delivery												
Vaginally without complications	330	62.5%	97	29.4%	233	70.6%	0.274	124	37.6%	206	62.4%	0.851
Vaginally with complications	64	12.1%	21	32.8%	43	67.2%		26	40.6%	38	59.4%	
Planned cesarean	38	7.2%	15	39.5%	23	60.5%		16	42.1%	22	57.9%	
Emergency cesarean	96	18.2%	37	38.5%	59	61.5%		40	41.7%	56	58.3%	
Gestation weeks												
28 to 37 weeks	25	4.7%	9	36.0%	16	64.0%	0.535	10	40.0%	15	61.2%	0.957
38 to 41 weeks	479	90.7%	151	31.5%	328	68.5%		186	38.8%	293	58.3%	
42 weeks or more	24	4.5%	10	41.7%	14	58.3%		10	41.7%	14	60.0%	
Birth weight												
More than 3 kg	383	72.5%	122	31.9%	261	68.1%	0.945	148	38.6%	235	61.4%	0.491
2–3 kg	139	26.3%	46	33.1%	93	66.9%		57	41.0%	82	59.0%	
1.5–2 kg	6	1.1%	2	33.3%	4	66.7%		1	16.7%	5	83.3%	
Feeding												
Feeding type												
Exclusive breastfeeding	339	64.2%	86	25.4%	253	74.6%	<0.001	119	35.1%	220	64.9%	0.045
Non-breastfeeding	52	9.8%	25	48.1%	27	51.9%		25	48.1%	27	51.9%	
Mixed	137	25.9%	59	43.1%	78	56.9%		62	45.3%	75	54.7%	
Feeding frequency												
Request	339	64.2%	86	25.4%	253	74.6%	<0.001	119	35.1%	220	64.9%	0.009
Fixed hours	189	35.8%	84	44.4%	105	55.6%		87	46.0%	102	54.0%	

SD—standard deviation; kg—kilograms. The significance level was set at *p* < 0.05. (^a^) Proportions were analyzed using the Chi-squared test.

## Data Availability

The data presented in this study are available upon request from the corresponding author. The data are not publicly available due to privacy and ethical restrictions.
